# The role of DNA methylation in placental development and its implications for preeclampsia

**DOI:** 10.3389/fcell.2024.1494072

**Published:** 2024-12-03

**Authors:** Yizi Meng, Yimei Meng, Linli Li, Yuan Li, Jin He, Yanhong Shan

**Affiliations:** ^1^ Department of Obstetrics, Obstetrics and Gynecology Center, The First Hospital of Jilin University, Changchun, China; ^2^ Department of Obstetrics and Gynecology, The Second Affiliated Hospital of Harbin Medical University, Harbin, China; ^3^ Department of General Gynecology I, Obstetrics and Gynecology Center, The First Hospital of Jilin University, Changchun, China

**Keywords:** preeclampsia, DNA methylation, placental development, epigenetic biomarkers, therapeutic targets

## Abstract

Preeclampsia (PE) is a prevalent and multifaceted pregnancy disorder, characterized by high blood pressure, edema, proteinuria, and systemic organ dysfunction. It remains one of the leading causes of pregnancy complications, yet its exact origins and pathophysiological mechanisms are not fully understood. Currently, the only definitive treatment is delivery, often requiring preterm termination of pregnancy, which increases neonatal and maternal morbidity and mortality rates, particularly in severe cases. This highlights the urgent need for further research to elucidate its underlying mechanisms and develop targeted interventions. PE is thought to result from a combination of factors, including inflammatory cytokines, trophoblast dysfunction, and environmental influences, which may trigger epigenetic changes, particularly DNA methylation. The placenta, a vital organ for fetal and maternal exchange, plays a central role in the onset of PE. Increasing evidence suggests a strong association between DNA methylation, placental function, and the development of PE. This review focuses on the impact of DNA methylation on placental development and its contribution to PE pathophysiology. It aims to clarify the epigenetic processes essential for normal placental development and explore potential epigenetic biomarkers and therapeutic targets for PE. Such insights could lead to the development of novel preventive and therapeutic strategies for this condition.

## 1 Introduction

Preeclampsia (PE) is a serious pregnancy complication characterized by hypertension and multi-organ dysfunction, posing life-threatening risks to both mother and fetus. Affecting approximately 3%–5% of pregnancies globally, PE accounts for around 60,000 maternal deaths and contributes significantly to preterm birth and neonatal complications each year. PE typically presents after the 20th week of gestation and is marked by hypertension and organ dysfunction, which can manifest as proteinuria, renal failure, liver impairment, and, in severe cases, complications such as hemolysis, thrombocytopenia, hepatic rupture, seizures, stroke, and death. Identified risk factors include a history of pregnancy complications, diabetes, obesity, and multiple gestations, with incidence rates ranging from 8% to 20% in twin pregnancies and 12%–34% in triplet pregnancies (Ma’ayeh and Costantine, 2020). Currently, the only definitive treatment is early delivery, often required preterm in severe cases to prevent life-threatening complications for both mother and fetus (MacDonald et al., 2022; Roberts et al., 2021). This highlights an urgent need to better understand the underlying mechanisms of PE and to develop targeted interventions that could reduce the reliance on preterm delivery.

The pathogenesis of PE is complex and not fully understood. A widely accepted theory is Redman’s “two-stage model” of PE. In the first stage, inadequate trophoblast invasion, increased trophoblast cell death, reduced invasiveness, poor remodeling of the uterine spiral arteries, and impaired placental development lead to decreased placental perfusion. Although this stage is asymptomatic, it predisposes the pregnancy to the second, symptomatic phase, characterized by placental oxidative stress and inflammation. During this phase, the syncytiotrophoblast (STB) releases soluble vascular endothelial growth factor receptor 1 (VEGFR1) and soluble endoglin (sENG), triggering a systemic inflammatory response in the mother (Pankiewicz et al., 2021; Vasconcelos et al., 2023). This model underscores the pivotal role of the placenta in PE development. In normal pregnancies, trophoblasts invade the uterine spiral arteries, disrupting the smooth muscle layer to promote vascular remodeling, which is essential for fetal nutrition. In PE, however, placental dysfunction is associated with ischemia, hypoxia, and oxidative stress (Ashraf et al., 2021), though the precise molecular pathways contributing to these abnormalities remain to be fully elucidated.

Epigenetic changes are heritable alterations that do not involve changes in the DNA sequence but can significantly influence gene expression, with profound effects on placental development. Increasing evidence has shown that epigenetic regulation (Andrawus et al., 2022), through mechanisms such as DNA methylation, histone modifications, and non-coding RNA expression, plays critical roles in the regulation of genes involved in placentation and the pathogenesis of PE. A review by Nelissen et al. emphasizes that these mechanisms are not only integral to normal placental function but are also strongly implicated in the development of PE (Nelissen et al., 2011). Among these mechanisms, DNA methylation is one of the most extensively studied and is considered a key epigenetic modification. DNA methylation involves the addition of a methyl group to the cytosine base in CpG dinucleotides, a process catalyzed by DNA methyltransferases (DNMTs) using S-adenosylmethionine (SAM) as the methyl donor. This modification plays essential roles in various biological processes, including embryonic development, regulation of gene transcription, maintenance of chromatin structure, and X-chromosome inactivation. Several studies have reported abnormal DNA methylation patterns in the placentas of PE patients (Cirkovic et al., 2020; Dias et al., 2022). These aberrations in normal epigenetic regulation are crucial for healthy placental function, contributing to the pathophysiology of PE. Although genetic mouse models have provided valuable insights into pregnancy complications, they face limitations in fully replicating the complex, multifactorial nature of PE. The heterogeneity of the disease, driven by the intricate interplay between maternal and fetal factors, makes it difficult to develop reliable models. Such complexity emphasizes the importance of alternative approaches, particularly epigenetic mechanisms such as DNA methylation, which can capture dynamic environmental influences on gene regulation in the placenta.

By focusing on DNA methylation, researchers can uncover novel regulatory pathways that genetic models may miss, offering new opportunities for understanding PE and identifying potential biomarkers and therapeutic targets. Therefore, this review will highlight recent advances in understanding the role of DNA methylation in placental development and its association with PE, with a focus on how these findings could improve early diagnosis, prevention, and therapeutic strategies for managing PE.

## 2 Overview of DNA methylation

DNA methylation is one of the most extensively studied epigenetic modifications, playing a critical role in regulating gene transcription, primarily through the formation of 5-methylcytosine (5 mC). This modification occurs when a methyl group is added to the 5th carbon atom of cytosine within CpG dinucleotides, a process catalyzed by DNA methyltransferases (DNMTs) (Mahmoud and Ali, 2019). In addition to 5 mC, DNA methylation can also occur at the N-6 position of adenine and the N-7 position of guanine, resulting in the formation of N6-methyladenine (N6mA) and 7-methylguanine (7 mG), respectively, each catalyzed by distinct DNMTs (Li et al., 2022; Thomas et al., 2013). DNA methylation is essential for normal cellular function, embryonic development, and genomic regulation, and is also implicated in the pathogenesis of various diseases, making it a critical area of research (Cao et al., 2021). In the human genome, CpG islands, regions rich in cytosine-phosphate-guanine sequences, are key sites of DNA methylation, with approximately 30,000 CpG islands identified. These regions are located in the promoter areas of 60%–70% of known genes, and 60%–80% of them are susceptible to methylation, highlighting their pivotal role in regulating gene expression (Kim and Costello, 2017; Ciechomska et al., 2019). Altered methylation patterns in CpG islands can lead to dysregulated gene expression, affecting cellular homeostasis, DNA integrity, and genomic stability.

DNA methylation processes can be broadly categorized into two types: *de novo* methylation and maintenance methylation. *De novo* methylation introduces methyl groups to previously unmethylated DNA, while maintenance methylation preserves the methylation patterns during DNA replication by copying the methylation from the parent strand to the newly synthesized strand (Wang et al., 2021). The enzymes responsible for these processes are DNMTs, including DNMT1, DNMT2, DNMT3A, DNMT3B, and DNMT3L. DNMT1 primarily facilitates maintenance methylation, ensuring the replication of existing methylation patterns. DNMT3A and DNMT3B are involved in *de novo* methylation, establishing new methylation marks during development. DNMT3L, although lacking catalytic activity, acts as a regulatory cofactor, supporting DNMT3A and DNMT3B in *de novo* methylation. While DNMT2 shares structural features with DNA methyltransferases, it primarily functions as an RNA methyltransferase, methylating cytosine residues within specific tRNAs, rather than modifying DNA (Sriraman et al., 2020).

DNA demethylation is the reverse process of methylation and can occur both passively and actively (Chakraborty and Viswanathan, 2018; Sergeeva et al., 2023). Passive demethylation occurs during DNA replication when newly synthesized DNA strands fail to inherit the methylation marks, leading to the gradual dilution of 5-methylcytosine (5 mC) through successive cell divisions. Active demethylation involves the enzymatic removal of methyl groups (Prasad et al., 2021). The Ten-Eleven Translocation (TET) enzymes, a family of dioxygenases, play a critical role in this process by oxidizing 5mc into 5-hydroxymethylcytosine (5 hmC), and further to 5-formylcytosine (5 fC) and 5-carboxycytosine (5caC) (Ross and Bogdanovic, 2019). This reaction requires iron (Fe(II)) as a cofactor and uses oxygen and α-ketoglutarate as substrates (Joshi et al., 2022). Notably, 5 hmC is not merely an intermediate in demethylation but can also function as a stable epigenetic mark with distinct regulatory roles in gene expression (Vasconcelos et al., 2023). Recent studies have revealed that 5 hmC is enriched in imprinted regions of the placenta, where it plays an important role in regulating key genes involved in placental and fetal development (Hernandez Mora et al., 2018). This highlights the significance of 5 hmC as both a transitional and stable modification within the epigenetic landscape of mammalian genomes.

DNA methylation and demethylation processes often occur in the same genomic regions, directing crucial developmental stages. In general, DNA methylation is associated with gene repression, while DNA demethylation is linked to gene activation (Deng et al., 2023). The interplay between these two processes maintains the dynamic balance necessary for normal development and cellular function. Additionally, recent research highlights the significance of DNA methylation defects beyond the placenta. For instance, the review by Smith et al. (2024) discusses the crucial roles that DNA methylation plays in mammalian development and its implications for various diseases, including cancers, neurological disorders, and metabolic syndromes. Dysregulation of DNA methylation can lead to aberrant gene expression, which may contribute to the development and progression of these conditions.

## 3 DNA methylation and placental development

The “Developmental Origins of Health and Disease” (DOHaD) hypothesis suggests that conditions during gestation are key determinants of lifelong disease risk, with the placenta playing a crucial role. Beyond mediating nutrient and waste exchange between mother and fetus, the placenta is also vital in shaping fetal development. Recent studies have highlighted the involvement of DNA methylation in placental development (Jedynak et al., 2022).

### 3.1 Placenta and placental cells

The placenta is a crucial organ at the maternal-fetal interface, facilitating nutrient uptake, waste removal, and gas exchange while also serving as a barrier against infections (Rosenfeld, 2021). Structurally, the human placenta is discoid, comprising both fetal components (e.g., the amnion and chorion frondosum) and maternal components, including the decidua basalis. The amnion is located on the fetal side, while the decidua basalis is on the maternal side, facing the uterine endometrium, with the umbilical cord inserted on the fetal side (Longtine and Nelson, 2011).

The chorion frondosum is the primary structural element of the placenta. After implantation of the late blastocyst on the relatively flat surface of the endometrium, trophoblast cells at the implantation site undergo extensive proliferation. These cells differentiate into two layers: an inner layer of cytotrophoblasts (CTBs), which are the proliferative cells, and an outer layer of syncytiotrophoblasts (STBs), which arise from the CTBs and carry out the functional roles of the placenta. These layers, together with an adjacent layer of extraembryonic mesoderm, form the chorion. In the chorion frondosum, nutrient-rich villi develop along the decidua, creating intervillous spaces that house the expanding chorionic villi. These villi are primarily composed of fibroblasts, mesenchymal cells, endothelial cells, immune cells, and fetal-placental blood vessels, and they represent the functional units of the placenta (Li et al., 2020). It is important to note, however, that trophoblast function and placental development are highly sensitive to pathological conditions such as preeclampsia and intrauterine growth restriction. Successful placental development depends on proper trophoblast invasion into the uterus and remodeling of the spiral arteries to ensure an adequate nutrient supply for both the placenta and the developing embryo. Even in normal pregnancies, subtle variations in trophoblast behavior, influenced by genetic and epigenetic factors, could have long-term impacts on fetal development.

### 3.2 Normal placental development

Normal development of the human placenta requires tightly coordinated interactions between the trophoblast lineage and the maternal endometrium, mediated by various cytokines, including growth factors and inflammatory signals produced by the endometrium. Placental development begins at the blastocyst stage with the trophoblast epithelium (TE). By day five post-fertilization, the zygote develops into a blastocyst, comprising an inner cell mass (ICM), which forms the fetus, and the TE, which gives rise to the placenta. Upon contact with the uterine epithelium, the TE triggers specific gene expression programs (Cindrova-Davies and Sferruzzi-Perri, 2022; Nakashima et al., 2021).

On day five post-fertilization, the polar TE attaches to the columnar epithelial cells of the uterine mucosa (Knöfler et al., 2019). Although direct observation of these initial interactions in humans is lacking, evidence suggests that blastocyst implantation occurs directionally, with the embryonic pole oriented toward the endometrium (Valles and Domínguez, 2006; Massimiani et al., 2019). By days six to seven, early pregnancy samples show the TE fusing to form an initial syncytium (Haram et al., 2020). As the endometrium encases the blastocyst, it transforms into the decidua, while significant remodeling occurs at the implantation site (Lopata, 1996).

Subsequently, the STBs expand, forming a protective layer around the blastocyst, supported by the underlying proliferative CTBs. By approximately day eight, the STBs begin to develop fluid-filled lacunae that rapidly expand in the following days. This process results in the formation of trabeculae between the STB layers and columns. Initially, the STBs engulf the decidual glands, which contribute to the fluid in the intervillous space. Concurrently, the CTBs proliferate beneath the STBs and extend into the trabeculae, leading to the development of the trophoblastic shell, which partitions TE into three distinct zones (A) the primary villous layer adjacent to the blastocyst cavity, (B) the intervillous spaces and trabecular system, and (C) the trophoblastic shell nearest to the endometrium, which serves as a precursor to the basal plate. This shell is fully formed by day fourteen, facilitating the migration of trophoblast cells into the decidua as extravillous trophoblasts (EVT) and initiating trophoblastic invasion. These cells penetrate the inner third of the myometrium and merge to form multinucleated giant cells.

**FIGURE 1:**
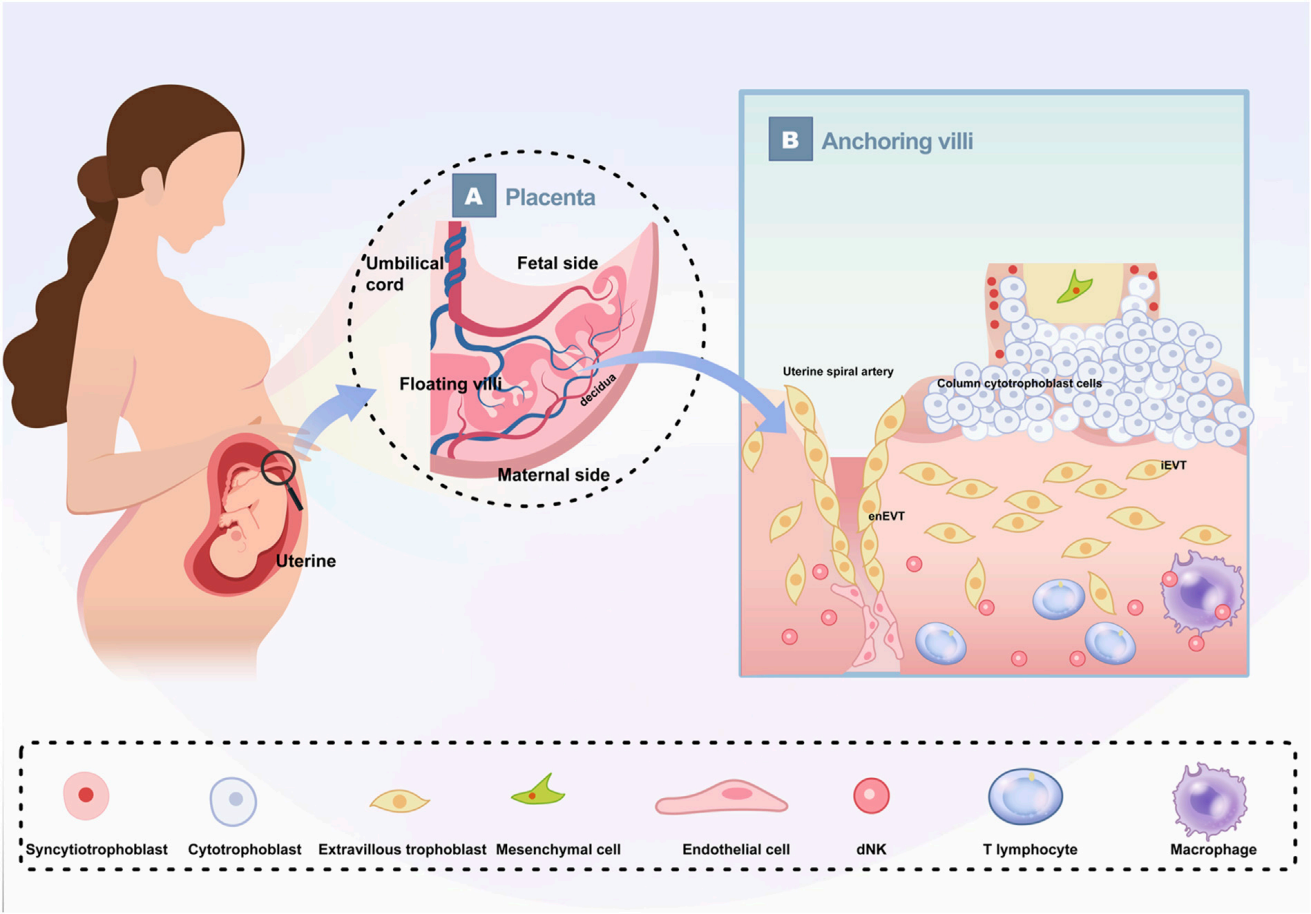
Schematic depiction of human placenta The growing fetus is connected to the uterus by the placenta **(A)** The basic structure of the placenta. The placenta is divided into maternal side and fetal side; The umbilical cord contains two umbilical arteries and one umbilical vein; The placental villi are divided into floating villi and anchoring villi. **(B)** Enlarged schematic diagram of anchoring villi and their cellular components.

Simultaneously, on day thirteen, CTB cells beneath the STBs proliferate vigorously, projecting into the cavity and branching, marking the beginning of primary villi formation. Shortly thereafter, mesenchymal cells infiltrate these villi, leading to the development of secondary villi. By day eighteen, the formation of tertiary villi commences as fetal capillaries emerge within the villous mesenchyme from angioblast progenitors derived from the mesenchymal cells (Ng et al., 2020; Zhao et al., 2021; Kojima et al., 2022; Covarrubias et al., 2023). The placental villi are categorized into anchoring villi and floating villi (Figure 1). The floating villi are immersed directly in maternal blood and primarily facilitate material exchange, while the anchoring villi secure the placenta to the uterus. Notably, when the spaces between villi are filled with maternal blood, the trophoblasts of the anchoring villi proliferate and stratify to form a highly dense column of cytotrophoblast cells (CCCs). The distal end of these CCCs can differentiate into EVTs, which migrate toward the uterine decidua. A subset of these extravillous trophoblast cells invades deeply into the endometrium and even into the myometrium’s outer third, anchoring the fetus within the maternal uterus, termed interstitial trophoblasts (iEVT). Another subset acquires endothelial-like characteristics, infiltrates the uterine spiral arteries, and replaces maternal endothelial cells, transforming these vessels into low-resistance, high-capacity uteroplacental arteries, known as endovascular trophoblasts (enEVT). This process, known as “spiral artery remodeling,” is necessary for establishing a robust maternal blood supply to the placenta, representing a crucial stage in the development of uterine-placental circulation (Apicella et al., 2019). By the end of the first trimester, the primary structures of the placenta are established, but their functionality may vary significantly depending on maternal health, environmental factors, and genetic predispositions.

### 3.3 Methylation regulatory mechanisms in placental development

Research shows that placental tissues exhibit lower methylation levels compared to other healthy tissues, but the underlying mechanisms and causes of this reduced methylation remain largely unknown. This phenomenon reflects the heterogeneity of placental tissue and the diverse methylation patterns across its different cellular populations. Comparative whole-genome methylation studies between human placentas and neutrophils (a more uniform cell type) indicate that placentas are generally hypomethylated, with an approximately 22% reduction in methylation levels. This decrease is observed in both intergenic regions and gene bodies, although promoter regions tend to remain unmethylated (Chatterjee et al., 2016).

Several studies have explored the origins of hypomethylation in placental tissues. After fertilization, the embryo undergoes extensive demethylation, followed by rapid *de novo* methylation within the ICM. In contrast, TE continues to exhibit low methylation levels (Smith et al., 2012; Robinson and Price, 2015). It is plausible that this persistent hypomethylation confers a selective advantage, potentially promoting the invasiveness of trophoblast cells, a critical feature for successful implantation and placental development. Moreover, research has implicated allele-specific downregulation of DNA methylation at DNMT1, a gene encoding DNA methyltransferase, in contributing to the global hypomethylation of trophoblast cells and chorionic villi (Novakovic et al., 2011). Research shows that placental tissues exhibit lower methylation levels compared to other healthy tissues, but the underlying mechanisms and causes of this reduced methylation remain largely unknown. This phenomenon reflects the heterogeneity of placental tissue and the diverse methylation patterns across its different cellular populations. Comparative whole-genome methylation studies between human placentas and neutrophils (a more uniform cell type) indicate that placentas are generally hypomethylated, with an approximately 22% reduction in methylation levels. This decrease is observed in both intergenic regions and gene bodies, although promoter regions tend to remain unmethylated.

Recent studies on mouse placentas have also examined the roles of DNMT3A, DNMT3B, and DNMT3L in gene regulation and development. Findings suggest that each DNMT enzyme targets specific chromatin features, contributing to distinct aspects of the placental methylome. Notably, the loss of DNMT3B led to the activation of germline genes in trophoblast cells and impaired the formation of the maternal-fetal interface, highlighting the crucial role of DNMT3B-mediated methylation in placental development and the survival of the embryo (Andrews et al., 2023). Furthermore, the hypomethylation observed in placental tissues is not uniform but localized within large regions known as partially methylated domains (PMDs). These PMDs, which cover about 40% of the placental genome, are also found in certain other tissues, such as cancer cells and some cultured cells, where DNA methylation is significantly reduced (Schroeder and LaSalle, 2013; Decato et al., 2020). These findings highlight the significant epigenetic variation across placental cell populations, though the functional implications of these differences for placental development remain incompletely understood.

As previously mentioned, the placenta’s complex cellular composition poses challenges for DNA methylation analysis. Most studies have focused on the chorionic villi, which are key sites for maternal-fetal exchange and hormone production, and display DNA methylation patterns distinct from those found in embryonic tissues, maternal decidua, or membranes such as the amnion and chorion (Bianco-Miotto et al., 2016). The chorionic villi consist of various cell types, including trophoblasts and ICM-derived cells, making them a central focus of research (Mayhew et al., 2004). A 2011 study sought to assess the cell-type specificity of DNA methylation in the placenta by isolating CTBs and fibroblasts from mid-pregnancy samples with high purity: 95% for trophoblasts and 60%–70% for fibroblasts. This study compared the methylation profiles of these isolated cells with those of the entire placental villi. Significant methylation differences were identified at 442 autosomal CpG sites between CTBs and fibroblasts, 315 sites between whole placenta and fibroblasts, and 61 sites between whole placenta and CTBs (Schroeder and LaSalle, 2013). These findings indicate substantial variability in DNA methylation across different placental cell types.

Research on trophoblasts is particularly important, as these cells play a critical role in remodeling the maternal vasculature to ensure sufficient blood flow and nutrient supply to the placenta. To investigate how genome-wide methylation differences influence trophoblast differentiation Gamage et al., (2018), conducted bisulfite sequencing on various trophoblast subtypes, including side population trophoblasts (potential human trophoblast stem cells), CTBs (intermediate progenitor cells), and extravillous trophoblasts (EVTs, terminally differentiated cells) from early pregnancy placentas. Their findings revealed distinct methylation patterns across these populations: side population trophoblasts exhibited a methylome most similar to that of CTBs, while EVTs displayed more distinct methylation profiles. Notably, side population trophoblasts showed differential methylation in genes and miRNAs associated with the cell cycle, differentiation, and pluripotency, including 41 genes involved in epithelial-to-mesenchymal transition, which may help explain the invasive nature of EVTs. However, the complex relationship between methylation and gene expression suggests that other regulatory mechanisms, such as demethylation, may also be crucial in placental development. Further exploring this Fogarty et al. (2015), employed quantitative immunohistochemistry to measure differences in 5 mc and 5 hmC levels between STBs and CTBs. Their results that CTBs had higher levels of 5mc, while STBs had elevated levels of 5 hmC. Although the biological significance of these differences is not completely defined, they highlight the considerable variability in methylation landscapes across different placental cell types.

Interestingly, DNA methylation levels differ between placentas of male and female fetuses, with notable variations in regions containing long interspersed nuclear elements (LINEs), Alu repeat sequences, and promoter CpG islands associated with X-linked genes (Bulka et al., 2023). Cotton et al. (2009), using DNA methylation arrays to examine X chromosome-associated promoter CpG islands, found lower methylation levels in female placentas compared to males, suggesting reduced methylation on the inactive X chromosome. Further analysis by Bianco-Miotto et al. (2016), examining 8,346 CpG sites on the X chromosome in placental tissues from 22 female and 19 male full-term pregnancies, confirmed that female placentas exhibit higher overall methylation levels than male placentas. This sex-based difference in methylation could have profound implications for understanding fetal development and the risk of sex-specific conditions.

DNA methylation during placental development plays a critical role in maternal and fetal health, with disruptions in methylation-regulated transcriptional processes linked to various diseases. Pearson et al. (2022) found that individuals with autism exhibited distinct CpG methylation patterns compared to healthy controls, with similar differences observed in placental tissues, suggesting possible effects on embryonic neural development. Additionally, variations in placental DNA methylation have been associated with differing risks of chronic lung disease (CLD) in extremely preterm infants Jackson et al. (2022). identified differential methylation at 49 CpG sites across 46 genes involved in fetal lung development pathways, such as the p53 signaling and inositol biosynthesis pathways, linking these variations to CLD incidence. Fetal growth restriction (FGR), which increases the risk of perinatal morbidity and mortality, has also been associated with alterations in DNA methylation Shi et al., (2023). conducted whole-genome methylation analyses on 24 placental samples from twelve FGR monochorionic twins using the Illumina Infinium Methylation EPIC BeadChip. They found a general trend of hypomethylation in placentas from growth-restricted fetuses, with pathway analysis revealing disruptions primarily in pathways related to steroid hormone biosynthesis, metabolism, cell adhesion, signaling, and immune responses.

Placental methylation abnormalities have significant implications for maternal health. For example, studies have identified elevated methylation levels at four CpG sites within a 264 bp promoter region of the placenta in cases of gestational diabetes, with these methylation changes correlating positively with glucose tolerance test results and negatively with lipoprotein levels, suggesting a potential role in the metabolic dysregulation observed in gestational diabetes (Chen et al., 2022). Additionally, research has uncovered notable differences in cytosine methylation and gene expression for 23 genes between women with recurrent miscarriages and healthy controls. Hi-MethylSeq analysis revealed increased methylation of SGK1 in both blood and decidual samples from women experiencing recurrent miscarriages, indicating a potential link to reproductive failure (Zhou et al., 2021). Furthermore, abnormal placental DNA methylation has been associated with other pregnancy complications, such as spontaneous abortion and preterm birth (Wang et al., 2022). These findings highlight the importance of understanding the placental epigenome in relation to these disorders, as it could offer new avenues for prevention and therapeutic intervention.

## 4 The role of methylation in the pathogenesis of PE

Previous studies have demonstrated that PE is an independent cardiovascular risk factor for mothers and has long-term adverse effects on the cardiovascular health of their offspring. For instance, children from PE-complicated pregnancies frequently exhibit elevated blood pressure, higher body mass index, and increased vascular stiffness compared to those from uncomplicated pregnancies. Although these clinical outcomes are well-documented, the underlying molecular mechanisms, including the role of DNA methylation, remain insufficiently explored (Brodowski et al., 2019). Anderson et al. (2014) conducted a genome-wide analysis of DNA methylation in peripheral blood cells and placental chorionic tissues from both normotensive and PE-affected women. They identified significant differences in methylation at 207 CpG sites, with 132 sites showing increased methylation and 75 sites showing reduced methylation. Similarly Yeung et al. (2016), identified 303 differentially methylated regions in PE placentas compared to controls, with most of these regions being hypermethylated. These regions were associated with genes involved in cell adhesion, Wnt signaling, and transcriptional regulation. A systematic review further highlighted lower overall methylation levels in placental tissues from PE patients, particularly in cases of early-onset and preterm PE (Cruz et al., 2020). These findings support the critical role of DNA methylation in the pathogenesis of PE and its associated complications.

### 4.1 The role of DNA methylation in trophoblast apoptosis

Maintaining a balance between trophoblast proliferation and apoptosis is crucial for the proper remodeling of uterine spiral arteries, and disruptions in this balance are associated with the development of PE. Several studies have implicated key regulatory molecules, such as miRNAs and transcriptional repressors, in mediating these processes through epigenetic changes. For example, it has been shown that high levels of let-7a expression in severe early-onset PE inhibit cell viability and cell cycle progression in JEG-3 cells, leading to increased apoptosis. Although this suggests a strong relationship between elevated let-7a levels and trophoblast apoptosis, the reliance on *in vitro* models (e.g., JEG-3 cells) raises concerns about the generalizability of these findings to *in vivo* conditions. JEG-3 cells, being derived from choriocarcinoma, may not fully recapitulate the behavior of normal trophoblasts, particularly in the complex environment of the placenta. Nonetheless, the demethylation of let-7a-3, which elevates let-7a expression and promotes apoptosis, suggests the involvement of DNA methylation in PE progression (Zha et al., 2020).

In addition, miR-155 is significantly upregulated in PE placental tissues and inversely correlated with its promoter methylation levels. miR-155 reduces trophoblast viability and enhances apoptosis, targeting the FOXO3 gene. Treatments with 5-Aza or co-transfection with si-FOXO3 have been shown to counteract these effects, indicating that methylation-mediated silencing of miR-155 can reduce trophoblast apoptosis by upregulating FOXO3 (Luo et al., 2022). Similarly, the overexpression of NSPc1 in PE trophoblasts, which accelerates apoptosis by repressing HOXA11 expression through interaction with DNMT3a, highlights another potential epigenetic mechanism involved in PE (Xie et al., 2023). Further complicating the picture is the role of hypoxia-induced promoter hypermethylation of ERO1α, which suppresses its expression, exacerbating endoplasmic reticulum stress and promoting trophoblast apoptosis. DNMT1, which increases its binding to the ERO1α promoter under hypoxic conditions, has been identified as a key regulator in this process, further linking DNA methylation changes to trophoblast apoptosis in PE (Xiong et al., 2018). Lastly, the involvement of other genes such as STAT5A (Rahat et al., 2016), SERPINA3 (Chelbi et al., 2012), and GATAD1 (Ma et al., 2014) in PE through methylation alterations provides additional mechanistic insights, but the functional relevance of these changes remains poorly understood.

### 4.2 The role of DNA methylation in trophoblast invasion and migration

Trophoblast invasion and migration are essential for proper placental development, playing a vital role in implantation and overall placental growth. Aberrations in these processes are closely linked to the pathogenesis of PE. A growing body of evidence suggests that DNA methyltransferases, particularly DNMT3A and DNMT1, as well as demethylating enzymes such as TET2, play pivotal roles in regulating trophoblast function and influencing PE development. Research has shown that aberrations in DNMT3A expression in PE placental tissues significantly impair trophoblast migration and invasion. Knocking down DNMT3A disrupts these crucial processes, largely through its impact on the expression of key regulatory genes. For example, the upregulation of IGFBP5, caused by promoter hypomethylation associated with reduced DNMT3A activity, negatively affects trophoblast function. However, suppressing IGFBP5 expression has been shown to mitigate the detrimental effects of DNMT3A knockdown (Jia et al., 2017). In addition to IGFBP5, reduced DNMT3A expression promotes activation of the TGFBR1 and TGF-β signaling pathways, which are key players in the pathophysiology of severe early-onset PE. Blocking TGFBR1 or inhibiting the downstream TGF-β/Smad pathway has been shown to reverse the impaired migration and invasion of trophoblasts, suggesting DNMT3A modulates PE development through multiple interconnected pathways (Jia et al., 2020). DNMT1 has also been implicated in the regulation of trophoblast function in PE. Elevated DNMT1 activity in PE placental tissues or trophoblast cell lines (e.g., HTR8/SVneo cells) has been associated with increased methylation of the APLNR promoter, reducing APLNR expression and consequently impairing trophoblast function. Interestingly, lowering DNMT1 levels reduces APLNR promoter methylation, leading to enhanced transcription of eNOS and improved trophoblast functions (Zhou et al., 2023). Furthermore, recent studies have shed light on the role of the demethylating enzyme TET2 in PE. A reduction in TET2 expression and the corresponding decrease in 5 hmC levels in PE placentas have been shown to impair trophoblast function. Specifically, TET2 downregulation leads to reduced expression of MMP9, an enzyme critical for tissue remodeling during placental development (Li et al., 2018). Another study revealed that ADAMTS7, a disintegrin and metalloproteinase with thrombospondin motifs, is upregulated in PE placentas, with its promoter exhibiting hypomethylation. Increased ADAMTS7 expression disrupts the functionality of trophoblast cell lines, such as HTR-8/SVneo and JEG-3 cells, implying that hypomethylation of this gene may contribute to the pathogenesis of PE (Zhang et al., 2020).

Although DNA methylation and demethylation are involved in the pathogenesis of PE, translating these molecular findings into effective therapeutic strategies presents both opportunities and challenges. Targeting key regulators such as DNMT3A, DNMT1, or TET2 shows considerable promise; however, the widespread effects of these enzymes on global gene expression necessitate a highly cautious and selective approach. Therapeutic interventions should prioritize precision, ideally focusing on specific genes or pathways that contribute to trophoblast dysfunction, while minimizing disruption to broader epigenetic regulatory networks.

## 5 Summary and outlook

In summary, DNA methylation changes have a profound impact on placental development and the progression of PE. While the field of epigenetics, especially regarding DNA methylation, is still evolving, it is clear that many methylation abnormalities associated with placental function and PE are yet to be discovered. The potential use of DNA methylation markers as diagnostic tools for PE shows promise but requires further validation. Additionally, investigating novel therapeutic approaches that target DNA methylation abnormalities could offer a new avenue for managing PE, presenting a promising research direction that may significantly improve patient outcomes.
